# Dispersive 2D Cherenkov radiation on a dielectric nano-film

**DOI:** 10.1038/s41598-017-06176-1

**Published:** 2017-07-19

**Authors:** Weihao Liu

**Affiliations:** National Synchrotron Radiation Laboratory, University of Science and Technology of China, Hefei, Anhui 230029 China

## Abstract

We report a modified two-dimensional Cherenkov radiation, which occurs on a high-index dielectric nano-film driven by uniformly moving electron-beam. It is essentially different from the ordinary Cherenkov radiation in that, in the nondispersive medium, it shows unique dispersion characteristics—the waves with higher frequencies radiate at larger Cherenkov angles. Its radiation frequency and direction are essentially determined by structure parameters as well as the beam-velocity. By means of fully electromagnetic simulations and theoretical analyses, we explored the mechanism and requirements of this radiation. This new Cherenkov radiation may lead to promising applications in a broad range of fields.

## Introduction

The Cherenkov radiation (CR), which is generated from uniformly moving charged particles with velocities being greater than the light speed in the background medium^1^, has been one of the most attractive research topics over the past decades^2^. It has been widely applied in particle detections and accelerations^3, 4^, beam diagnostics^5^, and radiation sources^6^ *etc*. Recent researches either explored the CR in new mediums, such as metamaterials^7, 8^ and photonic crystals^9^, or sought for more interesting properties, such as concentrating the CR in a finite region^10^ and considering the CR from short relativistic bunches in arbitrary slow-wave guiding systems^11^.

Most of previous investigations concerned cases that particles were surrounded by axisymmetric mediums with symmetric boundaries, such as three-dimensional (3D) infinite dielectrics and closed metallic waveguides. Recently, free-electron induced radiations from micro or nano-scale surfaces, such as the graphene^12^, metasurfaces^13^, and surface-plasmon films^14, 15^, have attracted increasing attentions: indicating promising developments of on-chip light sources and particle detectors. In the present paper, we will demonstrate a modified two-dimensional (2D) CR on a non-dispersive high-index dielectric (HD) nano-film. Compared with the traditional CRs in 3D space and other radiations, it shows unique and interesting dispersiveness and tunability, which may lead to new attractive applications.

## Results

**Figure 1:**
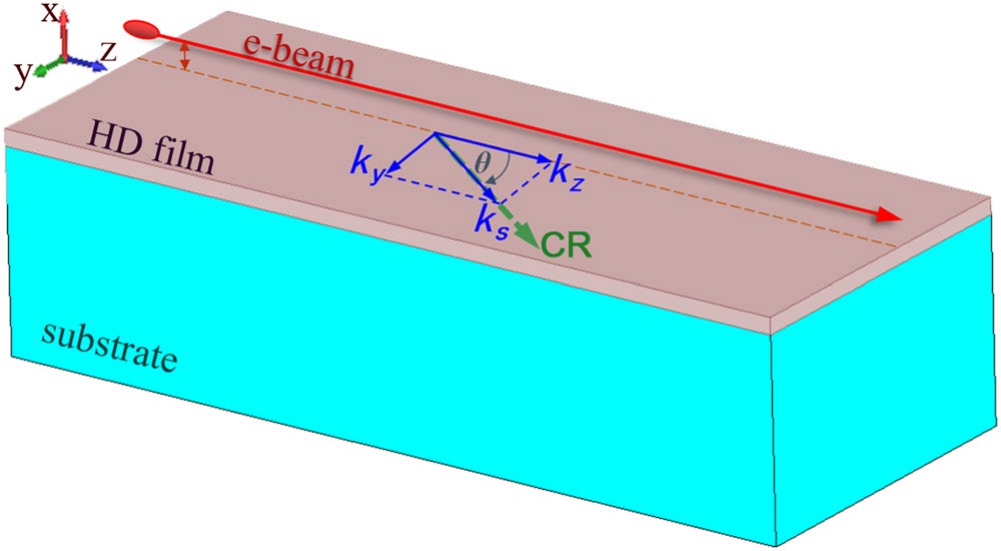
Schematic diagram of the free-electron exciting CR on a HD nano-film. The *k*_*s*_ is the total horizontal wave-vector of the CR on the nano-film, *k*_*y*_ and *k*_*z*_ are respectively wave-vectors in the y-direction and z-direction. *θ* denotes the propagation direction of the CR on the nano-film.

The schematic diagram of the physical model considered in the present paper is shown in Fig. 1. The HD nano-film is set up on an low-index dielectric substrate. And a uniformly moving free-electron-beam parallelly skims over the nano-film. We mainly consider the horizontally propagating waves along the nano-film. Both the nano-film and the substrate are assumed to be horizontally borderless, and the thickness of substrate is treated as infinite. In this scenario, the radiation happens on a 2D surface with unsymmetrical boundaries in 3D space.

**Figure 2:**
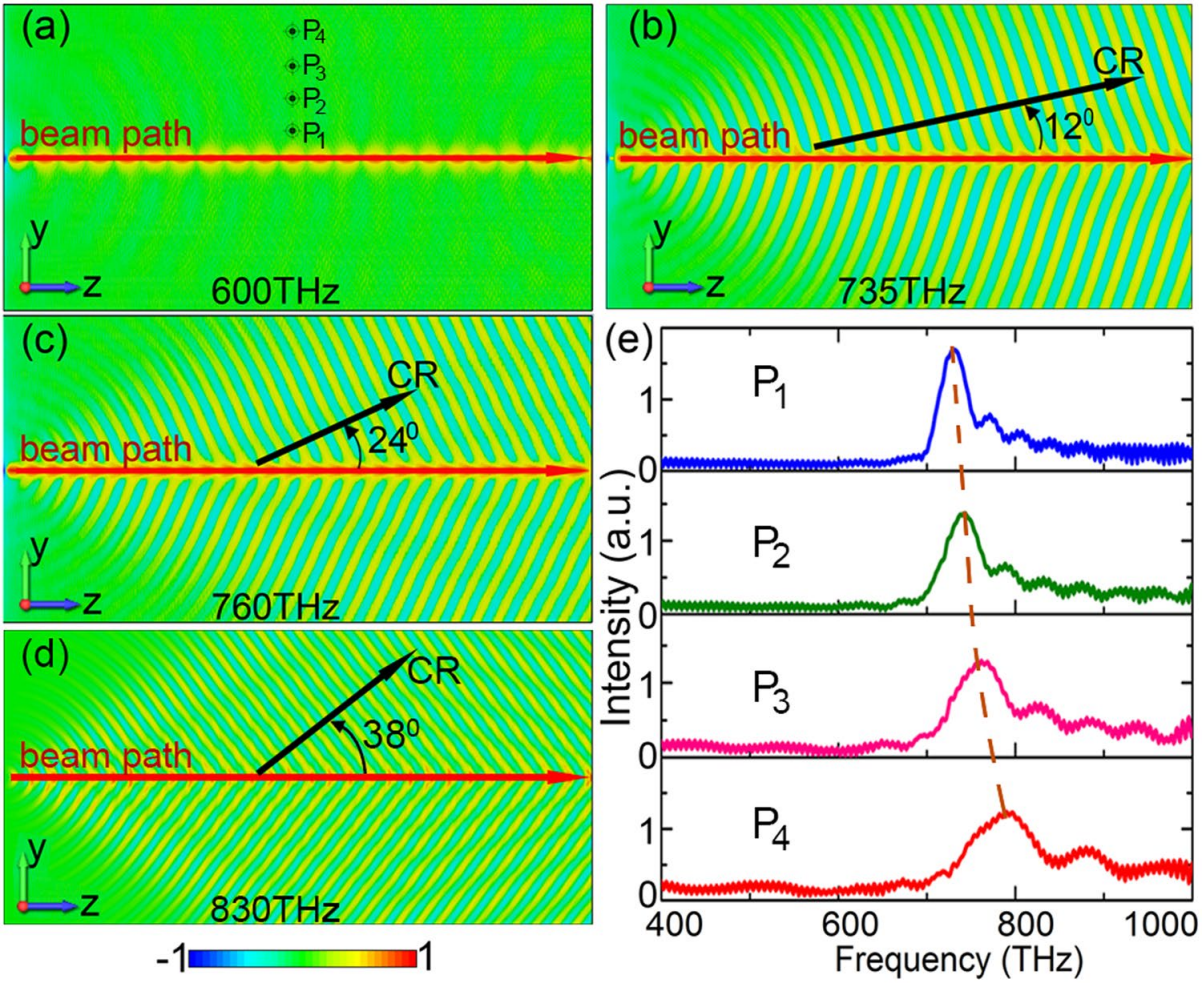
Simulation results of contour maps and spectra on the nano-film. (**a**–**d**) are contour maps of the *E*_*x*_ field on the surface of the nano-film for waves respectively with frequencies of 600 THz, 730 THz, 760 THz, and 830 THz. (**e**) The spectra detected at positions of ‘*P*_1_’–‘*P*_4_’ in Fig. 2(a).

**Figure 3:**
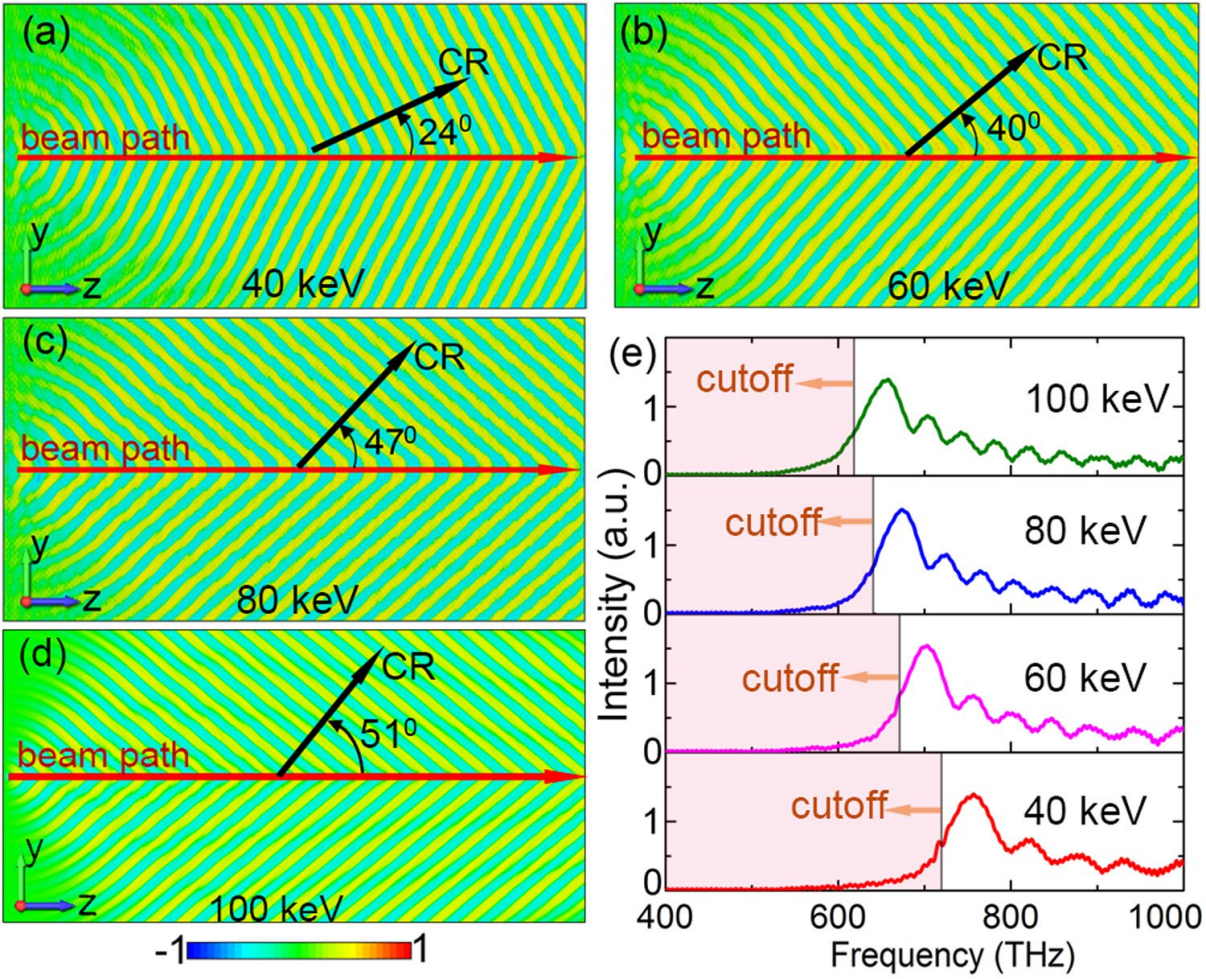
(**a**–**d**) are contour maps of the *E*_*z*_ field on the surface of the nano-film respectively for cases with beam energies of 40 keV, 60 keV, 80 keV, and 100 keV. (**e**) The detected spectra.

We would like to first show the simulation results obtained by the FDTD based fully electromagnetic particle-in-cell (PIC) codes^16, 17^. In simulations, a Gaussian electron-beam-pulse with definite charge quantity and electron-energy (velocity) is generated from a point emission source. Without loss of generality, we set the charge quantity to be 1.6 fC and the electron energy to be 40 keV. The dielectric indexes of the HD nano-film and the substrate are 30 and 2.25, respectively. Both of them are non-dispersive mediums. The film thicknesses is 40 nm. Simulation results are shown in Fig. 2, in which subplots (a)-(d) illustrate contour maps of the electric field (*E*_*x*_) on the nano-film for waves respectively with frequencies of 600 THz, 730 THz, 760 THz, and 830 THz. One can see that their propagation properties remarkably differ from each other. For the case of 600 THz, electromagnetic (EM) fields are bounded to the beam-moving-path, indicating that they are evanescent in side-directions and the horizontal transverse radiation has not been generated. Whereas for other three frequencies, EM waves can transversely propagate away from the beam-moving-path, signifying that the 2D horizontal radiations are generated on the nano-film. Radiation directions of 730 THz, 760 THz, and 830 THz waves are respectively *θ* = 12^0^, *θ* = 24^0^, and *θ* = 38^0^ (*θ* denotes the angle from the electron-beam direction to the radiation direction). That is, higher frequencies lead to greater radiation angles, indicating a unique dispersiveness. Figure 2(e) presents the frequency spectra of waves detected at different positions [points ‘*P*_1_–*P*_4_’ in Fig. 2(a)] on the nano-film. As the detection point moves away from the beam-moving-path, the radiation frequency increases gradually (blue shift). Changing electron-energy of the beam, we get simulation results shown in Fig. 3. Subplots (a–d) illustrate contour maps of *E*_*z*_ fields on the nano-film when electron-energy is 40 keV, 60 keV, 80 keV, and 100 keV, respectively. Their horizontal radiation directions are respectively *θ* = 24^0^, *θ* = 40^0^, *θ* = 47^0^, and *θ* = 51^0^ (here the frequency for all plots is 760 THz), which means that radiation angle increases gradually with beam-velocity. Figure 3(e) illustrates the field spectra (detected at point ‘*P*_4_’) for different beam-energies: the peak radiation frequency decreases as beam-energy increases. Thus, both radiation frequency and direction can be well controlled by adjusting the beam-energy (beam-velocity). We note that, in both Figs 2(e) and 3(e), all radiations show the high-pass spectra—only waves with frequencies higher than certain values can radiate on the nano-film.

## Methods

In order to understand above results and to explore mechanisms of these phenomena, we carry out theoretical analysis based on the basic electromagnetics. Detailed analytic derivations are performed in the supplement, in which the numerical calculated field contour maps agree well with the simulation results. Here we would like to summarize the main equations, presenting a physics picture of this radiation.

On the surface of the nano-film, the horizontal wave vector (*k*_*s*_) can be orthogonally decomposed into longitudinal component (*k*_*z*_) and transversal one (*k*_*y*_) (see Fig. 1), which leads to

1 $$\cos \,\theta ={k}_{z}/{k}_{s}\mathrm{.}$$

In the longitudinal direction, EM waves should be coupled by the electron-beam, indicating that

2 $$\omega /{k}_{z}={v}_{pz}={v}_{e},$$

in which *ω* is circular frequency, *v*_*pz*_ denotes the phase velocity of EM waves in the z-direction, and *v*_*e*_ is beam-velocity. Substituting Eq. (2) into Eq. (1) one gets

3 $$\cos \,\theta =\frac{\omega /{k}_{s}}{{v}_{e}}=\frac{{v}_{p}}{{v}_{e}},$$

in which *v*_*p*_ = *ω*/*k*_*s*_ denotes the total phase velocity of EM waves propagating along the nano-film. After examination, one can find that Eq. (3) exactly signifies a kind of CR with *θ* denoting the Cherenkov angle. To generate transverse radiation along the nano-film, the Cherenkov condition of *v*_*p*_ < *v*_*e*_ should be satisfied. Note that here the CR is on a horizontal 2D surface, and more interestingly it is a dispersive radiation (EM waves with different frequencies propagate in different directions as illustrated previously), which is essentially different from the traditional CR. For the traditional CR in 3D homogeneous mediums, EM waves with all frequency components radiate in the same direction (in the Cherenkov cone) and the radiation intensity is proportional to the square of frequency^18^. Only in dispersive (frequency-dependence) mediums can dispersive CR be generated. Thus a new kind of dispersive and tunable CR has been uncovered.

Now we are going to find out reasons of the dispersiveness and tunability of this CR. Based on the guided-wave theory, we know that frequency (*ω*) and wave-vector (*k*_*s*_) of the wave propagating along the nano-film should satisfy the dispersion equation:

4 $$F(\omega ,{k}_{s})=0.$$

**Figure 4:**
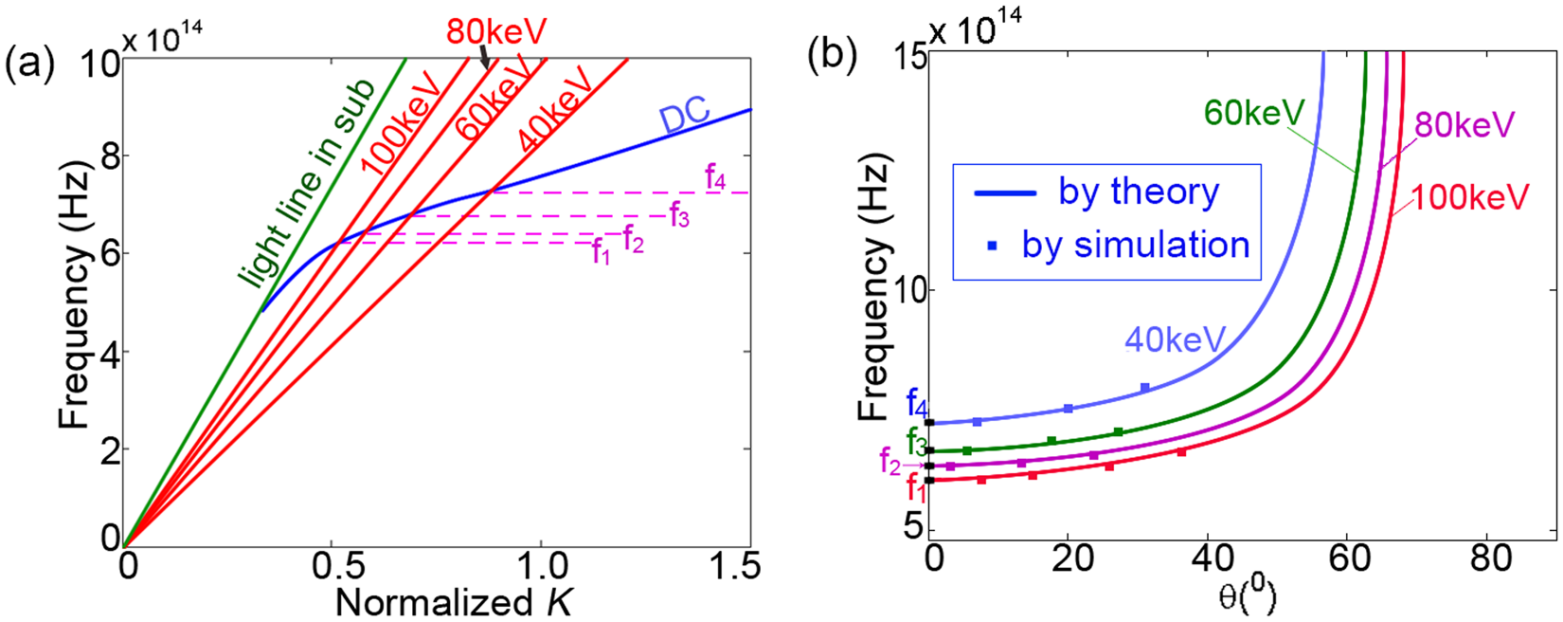
(**a**) Calculated dispersion curves of the nano-film and beam lines. (**b**) The frequency versus direction. The ‘${f}_{1}$’–‘${f}_{4}$’ are threshold (cutoff) frequencies respectively for beam energies of 40 keV, 60 keV, 80 keV, and 100 keV.

Combining Eqs (3) and (4), one can get:

5 $$\cos \,\theta =\frac{f(\omega )}{{v}_{e}},$$

in which *f*(*ω*) is the function derived from Eq. (4). In practices, *f*(*ω*) usually has not explicit expression because Eq. (4) is a transcendental equation which can only be solved numerically. Equation (5) exactly signifies the dependencies of the radiation direction ($\theta$) on frequency ($\omega$) and beam-velocity (${v}_{e}$), accounting for the dispersiveness of the 2D CR. Thus the radiation characteristics of the 2D CR are essentially determined by the dispersion relation of the nano-film: $F(\omega ,{k}_{s})$ or $f(\omega )$. For the HD nano-film considered in the present paper, $F(\omega ,{k}_{s})$ can be obtained by applying the mode-matching-method^14^:

6 $$F(\omega ,{k}_{s})=\frac{({\varepsilon }_{2}{k}_{1}-{k}_{2})}{({\varepsilon }_{2}{k}_{3}-{k}_{2}{\varepsilon }_{3})}{e}^{-{k}_{2}d}-\frac{({\varepsilon }_{2}{k}_{1}+{k}_{2})}{({\varepsilon }_{2}{k}_{3}+{k}_{2}{\varepsilon }_{3})}{e}^{{k}_{2}d},$$

in which ${k}_{1}=\sqrt{{k}_{s}^{2}-{k}_{0}^{2}}$, ${k}_{2}=\sqrt{{k}_{s}^{2}-{k}_{0}^{2}{\varepsilon }_{2}}$, ${k}_{3}=\sqrt{{k}_{0}^{2}{\varepsilon }_{3}-{k}_{s}^{2}}$, ${k}_{0}=\omega /c$, *c* is the light speed in vacuum, *d* is film-thickness, ${\varepsilon }_{2}$ and ${\varepsilon }_{3}$ are dielectric constants of the HD-film and the substrate, respectively. Numerically calculated dispersion curves are shown in Fig. 4, in which structure parameters follow that in Fig. 2. Beam-lines with different energies are also depicted. The dispersion curve in the right side of beam-lines denotes EM waves with phase velocities being less than beam-velocity (${v}_{p} < {v}_{e}$), indicating the Cherenkov condition is satisfied. The frequencies of these waves are higher than those of intersection points. In other words, only those EM waves with frequencies being higher than the threshold values can form the CR, which agrees with the high-pass feature illustrated in previous simulations. For the EM waves in the left side of beam-lines, the Cherenkov condition is not satisfied and the CR can not be generated, which accounts for the case of 600 THz in simulations of Fig. 2. Substituting Eq. (6) into Eq. (5), the radiation frequency versus direction is obtained as illustrated in Fig. 4(b). The frequency increases gradually with $\theta$, agreeing with the simulation obtained blue-shift shown in Fig. 2(e). The threshold frequency decreases gradually as beam-energy increases. For the same frequency, $\theta$ increases with beam-energy; while in the same direction, radiation frequency decreases with beam-energy. These results agree well with simulation results. Readily to know that the dispersion relation can be changed by adjusting structure parameters and dielectric constants of the nano-film and the substrate, indicating that the radiation properties of the 2D CR will be changed accordingly. Thus both the frequency and direction of the 2D CR will be well controlled.

Note that in all above cases, the beam-energies are not high enough to generate CR in the substrate, indicating that waves are evanescent in the substrate and the 2D CR can only propagate horizontally along the nano-film. We also note that all above 2D CRs were in the forward directions ($\theta {\mathrm{ < \; 90}}^{0}$). Predicably, the radiations can be steered into the backward directions ($\theta {\mathrm{ > \; 90}}^{0}$) provided that the HD material is replaced by left-handed metamaterials (LHMs), which is similar to the CR in the 3D LHMs^7^. The 2D LHM surfaces have been extensively investigated in previous studies^19^.

## Discussion

We would like to compare the modified 2D CR illustrated in the present paper with available radiations generated by uniformly moving charged particles. As demonstrated previously, it is obviously different from the ordinary CR in borderless 3D medium because of its dispersiveness and tunability. Another notable difference is that the ordinary CR expands, with the light speed in the medium, to the whole 3D space; contrarily, the 2D CR in the present paper is the guided-wave, which only propagates along the nano-film, and its phase velocity is less than the light speed in the vacuum or the substrate. In this sense, it is similar to CRs in the closed waveguides, such as dielectric loading waveguides (DLW) and periodic loading waveguides (PLW). In the closed waveguides, reflections from closed boundaries interfere with each other^20^, forming guided-modes in the waveguide. The CR in those scenarios is monochromatic radiation (with frequencies determined by operation points of guided modes) propagating exclusively in the longitudinal direction. Whereas for the 2D CR on the nano-film, the closed boundary reflections are eliminated, as a result of which the EM waves with different frequencies propagate in different directions instead of focusing in the forward or backward direction of the closed waveguide.

Then we compare the 2D CR with other kinds of free-electron-beam excited radiations, such as the Smith-Purcell radiation (SPR) and the transition radiation (TR), both of which are celebrated discoveries in the past century. The SPR is generated from charged particles passing over periodic surfaces^21^. Although both the SPR and the 2D CR are dispersive radiation from structures with open boundaries, the SPR is, in principle, the diffraction from periodic surfaces, which are indispensable for the SPR^22, 23^. Its radiation frequency decreases as radiation angle ($\theta$) increases^24–26^, which is just the opposite to the 2D CR of the present paper. As for the TR, occurring as charged particle passes through different mediums^27, 28^, it is caused by readjustment of EM fields at interface of adjacent media^29^. It will radiate into the whole 3D space, according to a certain angular distribution. Yet in the present paper, the electron-beam does not pass through or crash into the nano-film, and EM waves does not radiate into the whole 3D space. The 2D CR can be simply understood as below. When electron-velocity is greater than phase velocity of surface waves on the nano-film, these surface waves will be dumped by the electron-beam and propagate away from the beam-moving-path, which is a typical feature of CR^30^.

Now we discuss the possible applications of the 2D CR. As the radiation frequency and direction are well correlated with the beam-energy, it offers an effective way for the nondestructive detection of charged particles. Compared with the traditional CRs usually used for detection and diagnostic of high energy (relativistic) particles, the present CR can also diagnose sub-relativistic particles. Additionally it can be developed as a compact light source. Considering that the low energy beam can be generated by electron guns of scanning electron microscopes (SEMs), the present scheme is a promising table-top light source. Another probable application is for the manipulating and steering of light in the nano-scale structure, which are essential for the nanophotonics. Nanophotonics manipulate light in the subwavelength region beyond the diffraction limit. Previously, the surface plasmon polaritons (SPPs) on nano metallic structures had been widely studied^31–34^. Yet unfortunately, the practical applications of the SPPs are limited by the remarkable loss of metal^35^. The HD based structures can effectively avoid metallic loss, which enables them promising candidates for developing nanophotonics devices^35, 36^.

In conclusion, we have revealed a modified dispersive two-dimensional Cherenkov radiation on a high-index dielectric nano-film. It is essentially different from ordinary Cherenkov radiation and other kinds of radiations concerning the dispersiveness and tunability. The mechanism of this radiation has been found. This two-dimensional Cherenkov radiation may be of significance in physics and optics.

## Electronic supplementary material

Supplementary Information
