# Supplementary material for: Dispersive 2D Cherenkov radiation on a dielectric nano-film
Source: Sci Rep. 2017 Jul 19;7:5787. doi: 10.1038/s41598-017-06176-1 (PMC5517661; doi:10.1038/s41598-017-06176-1)
Supplement: Supplementary file 1 — Supplementary Information [file 41598_2017_6176_MOESM1_ESM.pdf]

Dispersive 2D Cherenkov radiation on a dielectric nano-film

Weihao Liu

National Synchrotron Radiation Laboratory, University of Science and Technology of China, Hefei, Anhui, 230029, China.

e-mail address: liuwhao@ustc.edu.cn

Supplement

In this supplement, analytic derivations of the two-dimensional dispersive Cherenkov radiation on the dielectric nano-film together with its dispersion relations are presented.

The diagram is shown in Figure A1. The whole space can be divided into three regions as shown in the inset. We will solve the Maxwell equations in different regions respectively, and then apply boundary conditions to get electromagnetic fields in all regions.

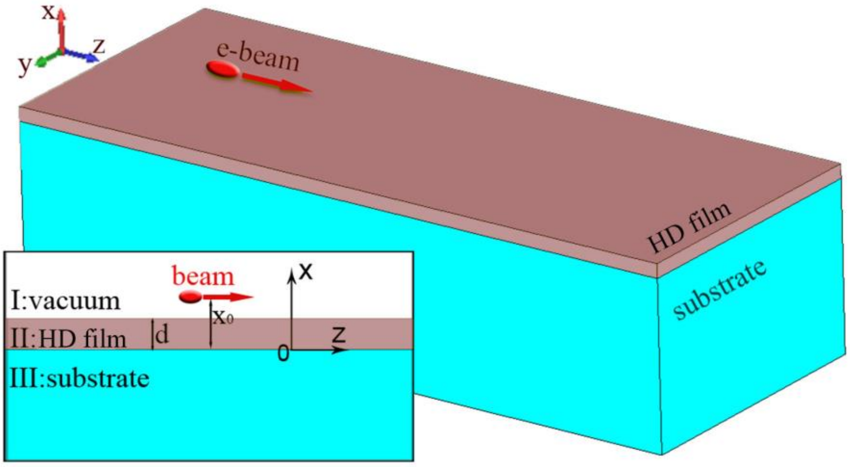

Figure A1. Schematic diagram.

According to the Maxwell equations, all other field components can be expressed in terms of the y-directional electric field component E_y and magnetic field component H_y, which will be considered at first.

In the upper half space (region-I shown in the inset of the figure), Fourier transformations of E^I_y and H^I_y components satisfy the following non-homogeneous equation in the Cartesian coordinate system shown in the figure[A1-A2]:

$$\begin{cases} \dfrac{\partial^2 E_y^I}{\partial x^2} + \dfrac{\partial^2 E_y^I}{\partial z^2} + (k_0^2 - k_y^2) E_y^I = -ik_y \dfrac{\rho(\omega)}{\varepsilon_0} \\ \dfrac{\partial^2 H_y^I}{\partial x^2} + \dfrac{\partial^2 H_y^I}{\partial z^2} + (k_0^2 - k_y^2) H_y^I = \mu_0 \dfrac{\partial J_z(\omega)}{\partial x} \end{cases} \tag{A1}$$

where $k_0 = \omega / c$, ω is angular frequency, c is light speed in vacuum, k_y is y-directional wave-number, ε_0 and μ_0 are respectively permittivity and permeability of the vacuum, $\rho(\omega)$ and $J_z(\omega)$ are Fourier transformations of charge density ρ and current density J, respectively. For particles with charge quantity of q and velocity of v, ρ and J can be expressed as:

$$\begin{cases} \rho = q\delta(x - x_0)\delta(y)\delta(z - vt) \\ J = qv\delta(x - x_0)\delta(y)\delta(z - vt) \end{cases} \tag{A2}$$

where δ is the Dirac delta function, x_0 denotes the beam position as shown in the figure. Substituting Eq. (A2) into Eqs. (A1), and applying the Fourier transformation method, E_y and H_y components in region-I can be solved. The solutions are composed by two parts: the special solution which represents the incident wave from particles and the general solution signifying reflection waves from the structure. Thus E_y and H_y fields in region-I can be expressed as:

$$\begin{cases} E_y^I = \dfrac{q}{2}\sqrt{\dfrac{\mu_0}{\varepsilon_0}}\dfrac{k_y k_z}{k_0 k_1} e^{ik_z z\text{-}k_1|x-x_0|} + A_1 e^{\text{-}k_1 x+ik_z z} \\ H_y^I = sign(x-x_0)\dfrac{q}{2} e^{-ik_z z\text{-}k_1|x-x_0|} + B_1 e^{\text{-}k_1 x+ik_z z} \end{cases} \tag{A3}$$

where $k_1 = \sqrt{k_z^2 + k_y^2 - k_0^2}$, $k_z = \omega/v$, $sign$ is the sign function, A_1 and B_1 are coefficients to be determined by boundary conditions.

The fields in region-II and region-III can be obtained by solving the homogeneous Helmholtz equations. In the HD nano-film (region-II with $0 \le x \le d$), the E_y and H_y components can be expressed as:

$$\begin{cases} E_y^{II} = A_2 e^{-ik_2 x+ik_z z} + A_3 e^{ik_2 x+ik_z z} \\ H_y^{II} = B_2 e^{-ik_2 x+ik_z z} + B_3 e^{ik_2 x+ik_z z} \end{cases} \tag{A4}$$

where $k_2 = \sqrt{\varepsilon_2 k_0^2 - k_z^2 - k_y^2}$, ε_2 is the dielectric constant of the nano-film, $A_{2,3}$ and $B_{2,3}$ are coefficients to be determined by boundary conditions. Here the waves reflected by both interfaces of the nano-film are considered.

In the substrate region (region-III with x<0), the E_y and H_y components can be expressed as:

$$\begin{cases} E_y^{III} = A_4 e^{k_3 x+ik_z z} \\ H_y^{III} = B_4 e^{k_3 x+ik_z z} \end{cases} \tag{A5}$$

where $k_3 = \sqrt{k_z^2 + k_y^2 - \varepsilon_3 k_0^2}$, ε_3 is the dielectric constant of the substrate, A_4 and B_4 are coefficients to be determined by boundary conditions. Note that waves in Eq. (A3) (region-I) and Eq. (A5) (region-III) are evanescent waves in the x-direction.

Making use of the Maxwell equations, all other field components can be expressed in terms of E_y and H_y by the following equations:

$$\begin{cases} E_t = \dfrac{-i}{k_o^2 - k_y^2}(k_y \nabla_t E_y + \omega\mu_0 \nabla_t \times H_y) \\ H_t = \dfrac{-i}{k_o^2 - k_y^2}(\omega\varepsilon_0 \nabla_t \times E_y + k_y \nabla_t H_y) \end{cases} \tag{A6}$$

Substituting Eqs. (A3~A5) to Eq. (A6), expressions of all field components in three regions can be obtained.

At boundaries of the nano-film, tangential electromagnetic fields satisfy following boundary matching conditions [A1,A3]:

$$\begin{cases} E_z^I\big|_{x=d} = E_z^{II}\big|_{x=d} \\ E_y^I\big|_{x=d} = E_y^{II}\big|_{x=d} \\ H_z^I\big|_{x=d} = H_z^{II}\big|_{x=d} \\ H_y^I\big|_{x=d} = H_y^{II}\big|_{x=d} \end{cases}, \begin{cases} E_z^{III}\big|_{x=0} = E_z^{II}\big|_{x=0} \\ E_y^{III}\big|_{x=0} = E_y^{II}\big|_{x=0} \\ H_z^{III}\big|_{x=0} = H_z^{II}\big|_{x=0} \\ H_y^{III}\big|_{x=0} = H_y^{II}\big|_{x=0} \end{cases} \tag{A7}$$

Substituting all tangential field components into Eq. (A7) and carrying out numerical calculations, the coefficients $A_{1,2,3,4}$ and $B_{1,2,3,4}$ can be solved, indicating that fields in all regions are obtained. When considering the 2D CR in the nano-film, we only need to get $A_{2,3}$ and $B_{2,3}$.

We note that in above equations all coefficients are determined by ω and k_y. Considering that k_y can be expressed as $k_y = k_z \tan\theta$, radiation fields essentially dependent on ω and θ. One of them is preset, we can get dependence of field on the other. Thus we can obtain the radiation intensity of the 2D CR depending on ω and θ, respectively. The numerical calculation results are shown in the following.

Calculated contour maps of the fields (E_y component) with different frequencies on the nano-film are shown in Figure A2, in which all parameters follow that given in Figure 2 of the main text. One can see that the radiation direction changes with frequency, agreeing well with simulation results presented in Figure 2 of the main text. It should be noted that all the calculated fields are in the frequency domain, such that the time evolution of particles skimming over the nano-film has not been illustrated.

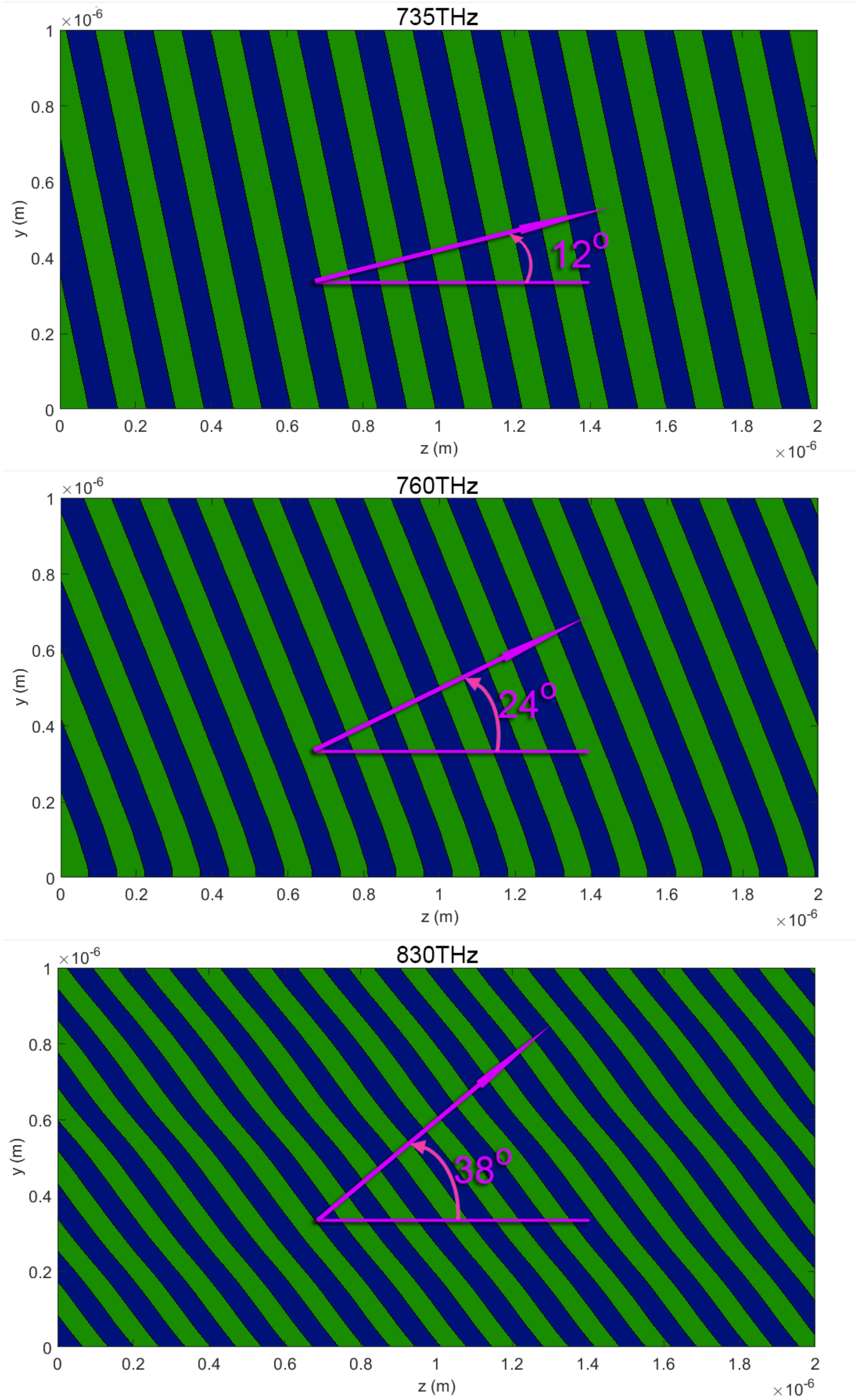

Figure A2. Calculated contour maps of the E_y field on the nano-film for different frequencies

Calculated contour maps of the fields (E_y component) with different electron-energies are shown in Figure A3, where all parameters follow that given in Figure 3 of the main text. One can see that the radiation direction changes gradually with beam-energy, being in well agreement with simulation results given in Figure 3

of the main text.

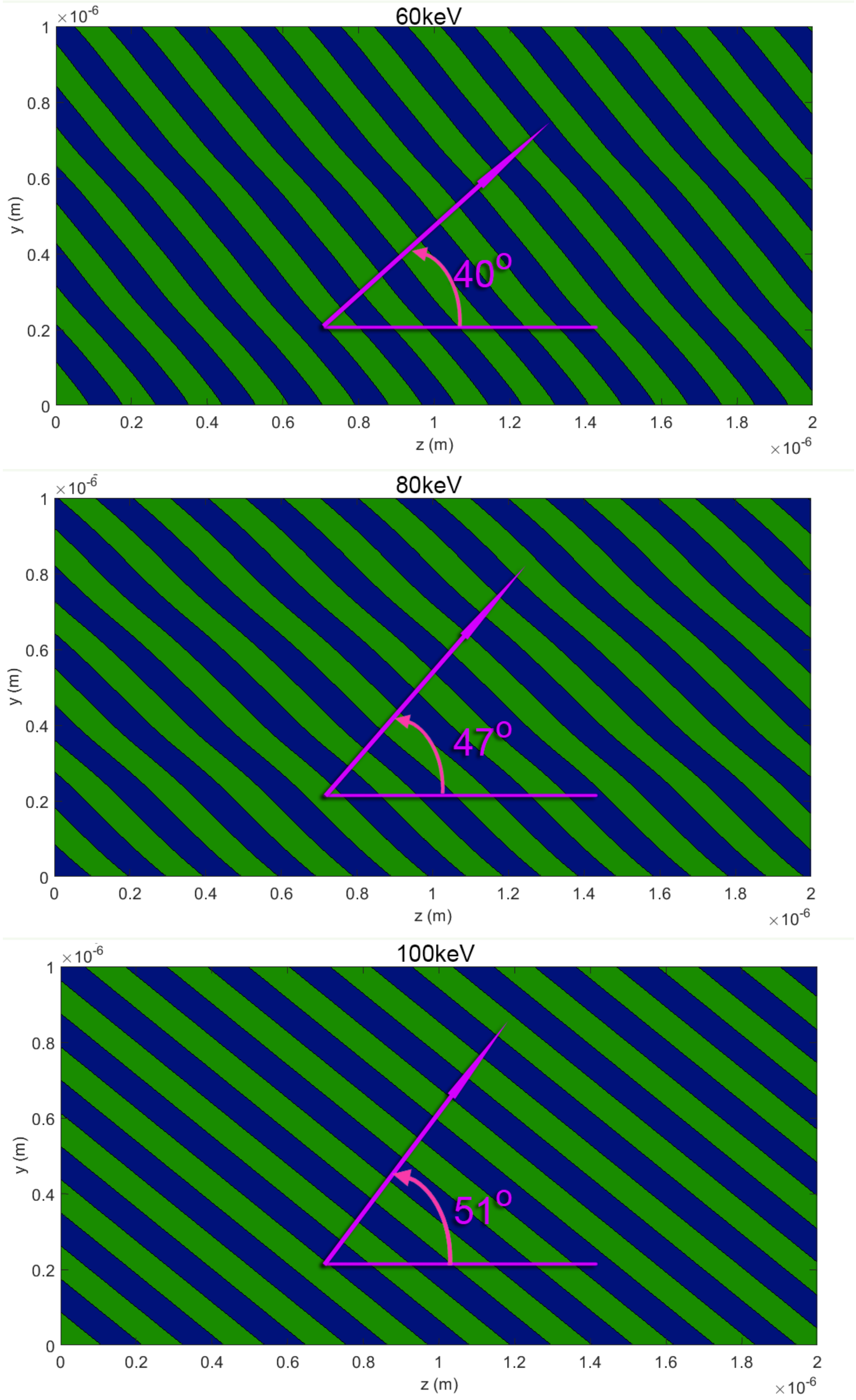

Figure A3. Calculated contour maps of the E_y field on the nano-film for different beam-energies

Now we consider the dispersion equation which governs wave propagating along the nano-film. We should solve the homogeneous equations (without particles) in all regions [A5]. Actually we only need to let the special solutions in Eq. (A3) to be zero,

keep field expressions in different regions presented above, and apply the boundary conditions of Eq. (A7). Then the dispersion equation, presented by Eq. (6) in the main text, can be obtained:

$$\frac{(\varepsilon_2 k_1 - k_2)}{(\varepsilon_2 k_3 - k_2\varepsilon_3)} e^{-k_2 d} = \frac{(\varepsilon_2 k_1 + k_2)}{(\varepsilon_2 k_3 + k_2\varepsilon_3)} e^{k_2 d} \qquad \text{(A8)}$$

where $k_1^2 = k_s^2 - k_0^2$, $k_2^2 = k_s^2 - \varepsilon_2 k_0^2$, $k_3^2 = k_s^2 - \varepsilon_3 k_0^2$, $k_s = \sqrt{k_y^2 + k_z^2} = \frac{\omega}{v_p}$, and v_p is phase velocity of wave along the nano-film. Equation (A8) can be numerically solved and dispersion curves of waves on the nano-film have been obtained as shown in Figure 4(a) of the main text.

References:

[A1] J. A. Kong, *Electromagnetic Wave Theory*, p195-p199, EMW Publishing, Cambridge, Massachusetts, 2000.

[A2] P. M. van den Berg, J. Opt. Soc. of Am., **63**, 689, 1973.

[A3] K. Zhang and D. Li, *Electromagnetic Theory in Microwave and Optoelectronics*, (Springer-Velag, Berlin, 2008).
